# Virtual Observation Units: A Novel Disposition for Older Adults With Falls From the Emergency Department

**DOI:** 10.1016/j.acepjo.2025.100230

**Published:** 2025-08-19

**Authors:** Angel Li, Scott A. Goldberg, Maura Kennedy, Emily Hayden, Kara Mc Loughlin, Kei Ouchi, Kalpana Shankar, Anita Chary, Shan W. Liu

**Affiliations:** 1 Department of Emergency Medicine, The Ohio State University Wexner Medical Center, Columbus, OH, USA; 2 Department of Emergency Medicine, Brigham and Women’s Hospital, Boston, MA, USA; 3 Department of Emergency Medicine, Massachusetts General Hospital, Harvard Medical School, Boston, MA, USA; 4 School of Allied Health, Ageing Research Centre, Health Research Institute, University of Limerick, Limerick, Ireland; 5 Department of Emergency Medicine, Baylor College of Medicine, Houston, TX, USA

**Keywords:** virtual observation units, geriatrics, falls, fall prevention, emergency department, older adults, virtual visits

## Abstract

Virtual observation units (VOUs) are being implemented across the country and serve as a model for patients to receive observational-level care at home. Falls are a leading cause of emergency department (ED) visits in the geriatric population and can cause substantial morbidity or mortality. Despite ED guidelines recommending fall-risk evaluation, the ED does not typically assess future fall risk given limited resources. We created a novel pilot VOU Falls Program that allows certain older ED fall patients to undergo fall-risk evaluation in their homes. In this concept paper, we discuss new care delivery models and how our VOU Falls Program integrates Mobile-Integrated Health paramedics, telemedicine emergency physicians, with fall-risk evaluation in the home. This novel VOU Falls Program includes home safety, functional testing, and fall-risk medication evaluations, components that are difficult to conduct in the ED, with the aim of reducing recurrent falls. It serves as an opportunity for emergency physicians to be at the forefront of a rapidly evolving area of care integrating Mobile-Integrated Health, telehealth for geriatric patients.

## 1 Background

Older adults make approximately 2.8 million emergency department (ED) visits annually.^1^ Falls are a leading cause of ED visits in the geriatric population with substantial associated morbidity or mortality,^2^^,^^3^ and over 30% of community dwellers aged >65 years fall at least once a year.^4^ Unintentional falls are the number one cause of death due to injury among older adults, with up to 20% of falls resulting in significant injury.^5^^,^^6^ Falls can lead to a decline in health, increased social isolation, nursing home admission, and loss of confidence.^6^ Further, over 1 in 5 patients may experience at least 1 subsequent fall within 6 months after a fall-related ED presentation.^7^

After a first fall, the ED encounter is an ideal opportunity to identify risk and limit the potential for future falls. However, despite ED guidelines recommending fall-risk evaluations,^8^ ED providers infrequently assess patients’ multifactorial risk for falls, in part because of time constraints^9^ and challenges in conducting assessments of mobility in the crowded ED environment. Therefore, the opportunity to identify patient risk and prevent future falls is missed.^10^^,^^11^

There has been a rapid growth of virtual home hospital services that maintain quality, safety, and patient experience,^12^, ^13^, ^14^, ^15^ catalyzed in part by a waiver from the Centers for Medicare and Medicaid Services providing a mechanism for reimbursement of home hospital services issued in response to the COVID-19 pandemic.^16^^,^^17^ Identifying the lack of fall-risk assessments in the ED, and leveraging our Mass General Brigham’s healthcare system’s experience with a hospital-at-home program, our ED developed a novel ED Virtual Observation Unit (VOU) Falls Program intended to address this gap in care for older patients with falls. Eligible older patients presenting to the ED after a fall are admitted to the VOU Falls Program, supported by telehealth and in-home evaluation by Mobile-Integrated Health (MIH) paramedics, after initial identification, screening, and enrollment from the ED. In this concept paper, we aim to describe the components and implementation of this program to assist other EDs that may be considering developing a VOU Falls Program to conduct fall-risk assessment and amelioration for their geriatric patients who have sustained a fall.

### 1.1 Clinical Management and Disposition of Older Fall Patients

Our hospital is a large, tertiary care academic medical center with an ED caring for 120,000 patients annually. It has a hospital-at-home program^12^^,^^14^^,^^16^ that can provide acute hospital-level care in a patient’s home (including in-person or virtual physician evaluations and a range of in-home diagnostics), as well as a traditional observation unit in close physical proximity to the ED. Any fall patients with significant injury or identified as high-risk for conditions such as syncope in the ED are typically admitted for inpatient care. Falls patients for whom ED clinicians are concerned about safety at home, eg, unable to ambulate independently to the bathroom, are often admitted to the observation unit in our hospital to await physical therapy (PT) consultation; some subsequently received postacute (rehabilitation) care.

Developed in an interdisciplinary collaboration involving emergency medicine physicians, geriatricians, paramedics, pharmacists, patients, nurses, and caregivers, we implemented the VOU Falls Program protocol in July 2022. Inclusion criteria for the VOU Falls protocol were patients aged ≥65 years presenting to the ED following a fall, determined by a clinician as being at risk for a subsequent fall, hemodynamically stable, and otherwise eligible for observation-level care. Patients residing in assisted living or a nursing home were not eligible.

### 1.3 Fall-Risk Assessment Components

The protocol incorporated evidence-based international guidelines on multifactorial fall-risk assessment for patients seeking medical attention due to a fall or recurrent falls,^18^ and includes a home safety evaluation, patient functional testing, and medication review (Figure).

**Figure:**
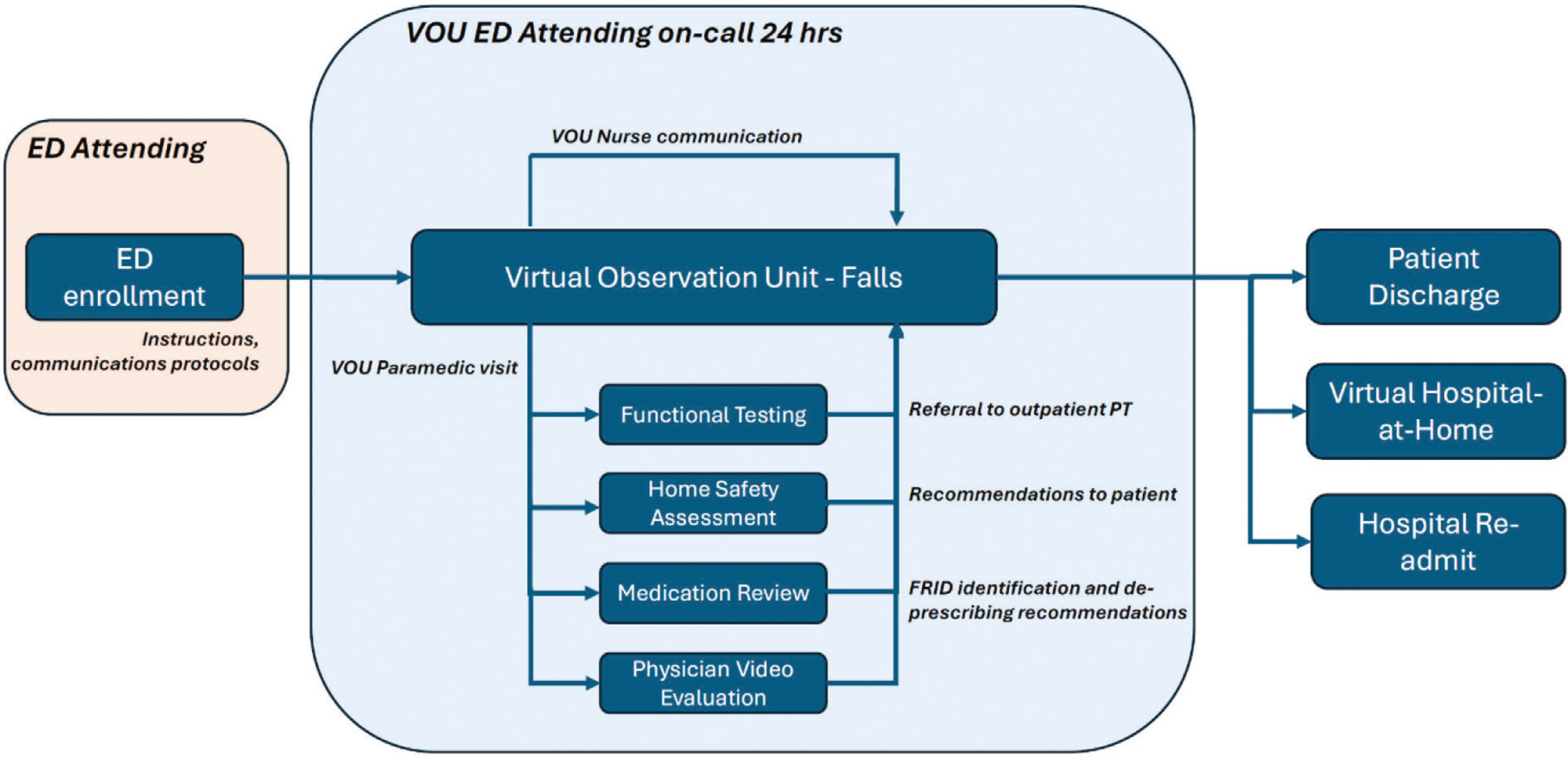
A novel virtual observation unit program for falls. VOU, virtual observation unit; ED, emergency department.

#### 1.3.1 Home Safety Evaluation

One important component of the VOU Falls Program is home safety evaluation, something that cannot be done when patients are admitted to the physical observation unit or even to the hospital. According to a Cochrane review by Gillespie,^4^ home safety assessment and modification interventions effectively reduced fall rates (risk ratio, 0.81; 95% CI, 0.68 to 0.97; 6 trials; 4208 participants) as well as the risk of falling (risk ratio, 0.88; 95% CI, 0.80 to 0.96; 7 trials; 4051 participants). In our program, MIH paramedics conduct a home safety evaluation when visiting the patient (Supplementary Appendix 1). They will report any concerns to the patient and telemedicine physician.

#### 1.3.2 Functional testing

Functional testing such as the Timed Up and Go (TUG) test predicts fall risk.^19^ Following the Centers for Disease Control and Prevention Stopping Elderly Accidents, Deaths, and Injuries algorithm,^20^ MIH paramedics use 2 cones and a 10-foot string to measure landmarks for the patient. Specifically, the telemedicine physician recommends PT for patients with a TUG test time greater than or equal to 12 seconds.^20^

#### 1.3.3 Medication review

Medication effects, side effects, and interactions are common causes of falls, and a number of fall-risk-increasing drugs (FRIDs) have been identified, including loop diuretics, opioids, antiepileptics, and benzodiazepines.^21^ A thorough medication review and reconciliation are recommended across fall prevention guidelines,^22^^,^^23^ and medication management and deprescribing are strongly recommended as a part of multifactorial fall-risk reduction intervention.^20^ Screening tools such as the Screening Tool of Older Persons Prescriptions in older adults with high fall risk^21^ and Screening Tool of Older Persons potentially inappropriate Prescriptions-Screening Tool to Alert doctors to Right Treatment^24^ can aid clinicians in drug-optimization strategies.

### 1.4 The ED VOU Fall Program

Participation in the VOU Falls Program is voluntary and by active consent of the patient or a legally authorized representative. After agreement, care is transitioned from the ED to the VOU Falls team. The patient or caregiver is provided instructions on the use of equipment (eg, tablets and pulse oximeter).

The VOU Falls team consists of the following members and roles: (1) VOU emergency medicine physician trained in fall risk assessments who visits virtually, (2) VOU nurse who communicates with patients twice a day, and (3) VOU MIH paramedic trained to provide out-of-hospital care in a nontransport capacity and supported by medical direction through telehealth who conducts a home visit and evaluation. The VOU nurse communicates with patients at designated scheduled times (typically morning and evening) for patient assessments via the computer tablet or telephone. A MIH paramedic with additional training specific to the VOU Falls protocol visits the patient at home within 24 h of admission to (1) conduct a safety evaluation of the home using a standardized checklist including stairs, lighting, bathtub or shower grab bars, and rugs (Supplementary Appendix 1); (2) assess mobility using the TUG test^19^; and (3) facilitate a video-based evaluation of the patient with the VOU emergency medicine physician. Medications are reviewed, and FRIDs are referred to the patient’s primary care provider for recommended deprescribing. Safety concerns are addressed with the patient and caregivers, including the importance of grab bars, floor rugs, and any other identified risks. Patients with an abnormal TUG are referred to outpatient PT.

Patients typically participate in the VOU Falls Program for 1 day. Although virtually all VOU patients are determined to be sufficiently safe to remain in their home following their VOU stay, any patient requiring continued care can be admitted to the virtual hospital at home or returned to the ED. We report a vignette here to illustrate the program.

**Potential Scenarios Following an Emergency Department Visit for a Fall**

JW, an 80-year-old female with congestive heart failure, anxiety, diabetes mellitus, and hypertension, presented to the ED after a fall. She reported that while ambulating to her kitchen she tripped, landing on her outstretched left hand. In the ED, she had discomfort of her left wrist but denied a head strike or cervical spine discomfort. No injuries were identified on plain films of the left wrist.

**Potential scenario 1**: She was discharged home. Two weeks later, she returned to the ED after a recurrent fall and was found to have a fracture of the right femoral neck.

**Potential scenario 2**: She was transferred home to participate in the VOU Falls Program. A trained visiting paramedic identified fall risks including area rugs and lack of shower grab bars, conducted a TUG mobility assessment test, and made a community referral for grab bar installation. After a telehealth consultation, a physician discussed TUG results with her and referred her to PT. The physician also emailed her primary care physician about the possibility of deprescribing 2 identified fall-risk-increasing drugs. She subsequently attended PT and has no recurrent fall in the next 6 months.

### 1.5 Advantages of the VOU Fall Program

We have identified a number of advantages of the VOU Falls Program. The provision of care in the home allows for distinct advantages over traditional hospital-based ED and observation unit care for patients who otherwise do not require hospitalization. A VOU allows for earlier care transitions and improved opportunities for medication reconciliation and deprescribing in coordination with the patient’s care team. Although based on the hospital-at-home model, these patients otherwise would not qualify for hospital admission. Further, a VOU provides an opportunity for more patient-centered care, treating the patient in the comfort of their own home; patients also perceive VOU care to be of high quality.^25^ With specific regard to postfall care, a VOU allows a more holistic evaluation of patient needs, including mitigation of risks present in the patient’s home, that would not be possible with traditional observation unit care.

Because the entire care delivery model occurs in the home, care transitions happen earlier, and clinicians can visualize how older adults can undertake care management activities in their own homes. This model allows clinicians to better understand care preferences, patient engagement, safety in the home setting, patient’s self-monitoring, social supports, and caregiver needs. Additionally, the VOU is ideally suited for identification of FRIDs as the patient is able to directly collect their home medications for evaluation. Opportunities for an accurate evaluation of FRIDs are limited within the ED or hospital settings.

Finally, the VOU Falls Program takes advantage of a growing group of healthcare providers and MIH providers. The MIH paramedics are becoming an increasingly important component of acute care at home programs, complementing more traditional role groups such as physicians and nurses. MIH is the provision of healthcare using patient-centered, mobile resources in the out-of-hospital environment in a coordinated manner with physicians, hospitals, and other healthcare and public safety providers such as emergency medical service (EMS) personnel. Although MIH programs may leverage a variety of clinical role groups, most MIH programs use paramedics with advanced training and an expanded scope of practice to provide out-of-hospital care in a nontransport capacity, often supported by medical direction through telehealth. EMS scope of practice is defined by the National EMS Scope of Practice Model,^26^ yet with appropriate training, oversight, and regulatory authority, this scope of practice can and should be expanded to meet the unique needs of specific populations and communities. Such an expanded scope of practice is safe, effective, and may result in substantial cost savings.^26^, ^27^, ^28^

MIH programs have already started to address fall prevention, although not specifically acute postfall recovery. In 1 randomized trial investigating home hazard assessment and removal, older patients randomized to an MIH program achieved a 38% reduction in the risk of falling.^29^ In another study, transport was successfully avoided in 65% of ground-level falls using a paramedic protocol and safety assessment, with a very low risk (0.3%) of missed pathology.^30^ However, the impact of fall-risk mitigation on patient-centered outcomes is less clear. A recent large multicenter trial in the United Kingdom randomized paramedics to usual care or to management at home with referral to a community falls service.^31^ Although subsequent EMS calls decreased in the intervention group, there was no identified change in emergency admissions, ED attendances, emergency service calls, or death.

## 2 Discussion

In the development of a novel VOU Falls Program, we have identified numerous advantages over traditional hospital-based observation care. This unique subset of patients does not qualify for inpatient-level care and, as such, would not qualify for a traditional hospital-at-home program. Patients admitted to a VOU may not require the full capabilities or length of stay as a patient admitted to a hospital-at-home program, but nevertheless require additional care beyond the immediate capabilities of the ED. The specific focus on this population of older adults at higher risk for subsequent falls, coupled with the additional training and systematic approach of our protocol, differentiates this program from existing VOU programs. By providing an in-home evaluation in the patient’s native environment, the VOU Falls Program provides safer transitions of care than can be offered in a traditional ED-based observation unit. VOU paramedics are well suited to help the telemedicine physician evaluate not only the patient’s medications but also the environment in which the medications are administered. This can help with medication reconciliation and deprescription, both advantageous in mitigating the deleterious effects of certain medications. Our systematized approach to the TUG test ensures functional testing can be done, something often not practically done in the ED due to a lack of space.

### 2.1 Future Directions

Future larger, randomized studies determining if a decrease in falls and ED revisits as well as the cost-effectiveness of the VOU program are needed. Furthermore, it would be beneficial to determine if fall patients who may need rehabilitation and placement could instead be accommodated in a VOU Falls Program. This approach could potentially open up physical observation unit beds and, thereby, alleviate ED crowding.

## 3 Conclusion

Despite the cost, morbidity, and mortality associated with falls among older adults, fall screening in the ED is difficult to implement because of time and resource constraints, resulting in potentially avoidable inpatient or observation admissions. VOUs provide an opportunity for a multifactorial fall-risk assessment and implementation of a fall mitigation plan performed in the patient’s home, with potential benefits in healthcare expenditures, hospital throughput, patient satisfaction, and reduced risk of subsequent falls. Although further outcome data are needed, our novel VOU program might serve as a model for other hospitals to develop similar programs.

## Funding and Support

By *JACEP Open* policy, all authors are required to disclose any and all commercial, financial, and other relationships in any way related to the subject of this article as per ICMJE conflict of interest guidelines (see www.icmje.org). The authors have stated that no such relationships exist.

## Conflict of Interest

All authors have affirmed they have no conflicts of interest to declare.
