# Supplementary material for: Virtual Observation Units: A Novel Disposition for Older Adults With Falls From the Emergency Department
Source: J Am Coll Emerg Physicians Open. 2025 Aug 19;6(5):100230. doi: 10.1016/j.acepjo.2025.100230 (PMC12396014; doi:10.1016/j.acepjo.2025.100230)
Supplement: Supplementary Appendix 1 [file mmc1.pdf]

Appendix: Home safety checklist

HOME SAFETY ASSESSMENT CHECKLIST

Patient Name: ___________________________ MRN: _____________________

Paramedic Name: _________________________ Date of visit: ________________

OUTSIDE OF HOME

1. Residence building is (circle one):	Single Family		Multi Family
2. Residence building is (circle one):	Single Floor		Multi-Floor
3. Sidewalk and/or pathway to house is level and free from any hazards:	Yes	No	N/A
4. Driveway is free from debris/snow/ice:	Yes	No	N/A
5. Outside stairs are stable and have sturdy handrail:	Yes	No	N/A
6. Porch lights are working and provide adequate lighting:	Yes	No	N/A
7. Elevator is in working order:	Yes	No	N/A

INSIDE HOME

1. Patient lives alone:	Yes	No	N/A
2. Patient has a pet that could potentially trip patient:	Yes	No	N/A
3. Patient has a pet/service animal that may confront visitors:	Yes	No	N/A

LIVING ROOM

1. Furniture is adequate height with supports to assist getting up and down:	Yes	No	N/A
2. Floor is free from clutter that would create tripping hazards:	Yes	No	N/A
3. All cords are secured and do not pose tripping hazards:	Yes	No	N/A
4. All rugs are secured to floor:	Yes	No	N/A
5. Lighting is adequate to light room:	Yes	No	N/A
6. All lighting has an easily accessible on/off switch:	Yes	No	N/A
7. Phone is readily accessible and chargeable near favorite seating areas:	Yes	No	N/A
8. Emergency numbers are available near all phones in house:	Yes	No	N/A
9. Fireplace has a cover:	Yes	No	N/A

KITCHEN

1. Items used more often are within easy reach:	Yes	No	N/A
2. Step stool is present, is sturdy, has handrail for higher shelves:	Yes	No	N/A
3. Floor mats are non-slip tread and secured to floor:	Yes	No	N/A
4. Oven controls are within easy reach:	Yes	No	N/A
5. Kitchen lighting is adequate and easy to reach switches:	Yes	No	N/A
6. ABC fire extinguisher is easily accessible:	Yes	No	N/A

STAIRS

1. Carpet is properly secured to stairs and/or all wood is properly secured:	Yes	No	N/A
2. Handrail is present and sturdy:	Yes	No	N/A
3. Stairs are free from any clutter:	Yes	No	N/A
4. Stairway is adequately lit:	Yes	No	N/A

BATHROOM

1. Tub and/or shower have a non-slip surface:	Yes	No	N/A
2. Tub and/or shower have a grab bar for stability:	Yes	No	N/A
3. Tub and/or shower is easy for patient to enter:	Yes	No	N/A
4. Toilet has nearby grab bar for assistance:	Yes	No	N/A

5. Pathway to bathroom is free from clutter and well lit: Yes No N/A

BEDROOM

1. Floor is free from clutter: Yes No N/A
2. Light is near bed and is easy to turn on: Yes No N/A
3. Phone is next to bed and within easy reach: Yes No N/A
4. Cell phone charger is next to bed and within easy reach: Yes No N/A
5. Flashlight is near bed in case of emergency: Yes No N/A

GENERAL

1. Smoke detectors in all areas of the house (each floor) and tested: Yes No N/A
2. CO detectors on each floor of house and tested: Yes No N/A
3. Flashlights are handy throughout the home: Yes No N/A
4. Resident has medical information readily accessible: Yes No N/A
5. All heaters are away from flammable materials: Yes No N/A

OVERALL TIPS

1. Patient has good non-skid shoes to move around house: Yes No N/A
2. All assisted-walking devices are readily accessible and in good condition: Yes No N/A
3. Home has clear pathways for wheelchair access if needed: Yes No N/A
4. There is a phone near the floor for ease of reach in case of a fall: Yes No N/A
5. All O2 tubing is less than 50 feet and is not a trip hazard: Yes No N/A
6. Resident has had an annual hearing and vision check: Yes No N/A
7. Resident has the proper hearing and visual aids in working order: Yes No N/A
8. All medications are properly stored and labeled with dosage times: Yes No N/A

For any statement marked "NO", the following recommendations and referrals are noted below:

____
____
____
____
____

Signature of Resident: ______________________________ Date of Assessment:______________

Signature of Paramedic: ______________________________ Time of Assessment:______________
